# Risk Factors of Microbial Keratitis in Uganda: A Case Control Study

**DOI:** 10.1080/09286586.2019.1682619

**Published:** 2019-10-22

**Authors:** Simon Arunga, Guyguy M. Kintoki, Stephen Gichuhi, John Onyango, Bosco Ayebazibwe, Rob Newton, Astrid Leck, David Macleod, Victor H. Hu, Matthew J. Burton

**Affiliations:** a International Centre for Eye Health, London School of Hygiene & Tropical Medicine, London, UK; b Department of Ophthalmology, Mbarara University of Science and Technology, Mbarara, Uganda; c Department of Ophthalmology, University of Nairobi, Nairobi, Kenya; d Ruharo Eye Centre, Ruharo Mission Hospital, Mbarara, Uganda; e Department of Epidemiology, Uganda Virus Research Institute, Entebbe, Uganda; f Tropical Epidemiology Group, London School of Hygiene & Tropical Medicine, London, UK

**Keywords:** Microbial keratitis, keratitis, diabetes mellitus, HIV, blindness, Uganda

## Abstract

**Purpose:**

Microbial keratitis (MK), is a frequent cause of sight loss worldwide, particularly in low and middle-income countries. This study aimed to investigate the risk factors of MK in Uganda.

**Methods:**

Using a nested case control, we recruited healthy community controls for patients presenting with MK at the two main eye units in Southern Uganda between December 2016 and March 2018. Controls were individually matched for age, gender and village of the cases on a 1:1 ratio. We collected information on demographics, occupation, HIV and Diabetes Mellitus status. In STATA version 14.1, multivariable conditional logistic regression was used to generate odds ratios for risk factors of MK and a likelihood ratio test used to assess statistical significance of associations.

**Results:**

Two hundred and fifteen case-control pairs were enrolled. The HIV positive patients among the cases was 9% versus 1% among the controls, *p* = .0003. Diabetes 7% among the cases versus 1.4% among the controls, *p* = .012. Eye trauma was 29% versus 0% among the cases and controls. In the multivariable model adjusted for age, sex and village, HIV (OR 83.5, 95%CI 2.01–3456, *p* = .020), Diabetes (OR 9.38, 95% CI 1.48–59.3, *p* = .017) and a farming occupation (OR 2.60, 95%CI 1.21–5.57, *p* = .014) were associated with MK. Compared to a low socio-economic status, a middle status was less likely to be associated with MK (OR 0.29, 95%CI 0.09–0.89, *p* < .0001).

**Conclusion:**

MK was associated with HIV, Diabetes, being poor and farming as the main occupation. More studies are needed to explore how these factors predispose to MK.

## Background

Microbial keratitis (MK), or infection of the cornea, can be caused by a range of pathogens. The causative organisms include bacteria, viruses, protozoa (e.g. acanthamoeba), and fungi (yeasts, moulds and microsporidia). It is characterised by acute or sub-acute onset of pain, conjunctival hyperemia and corneal ulceration with a stromal inflammatory cell infiltrate. MK frequently leads to sight-loss from dense corneal scarring, or even loss of the eye, especially when the infection is severe and/or appropriate treatment is delayed.^1^

MK in low and middle-income countries (LMIC) has been described as a “silent epidemic”, which leads to substantial morbidity, related to blindness and other consequences such as pain and stigma.^2^ It is the leading cause of unilateral blindness after cataract in Tropical regions and is responsible for about 2 million cases of monocular blindness per year.^3^ The World Health Organization (WHO) estimated (2017) that 1.3 million individuals are bilaterally blind from corneal opacity globally (excluding trachoma and vitamin A deficiency), accounting for 3.2% of binocular blindness.^4^ In Sub-Saharan Africa (SSA), MK is an important cause of binocular blindness and is responsible for about 15% of monocular blindness (Nigeria National Survey).^5,6^ The incidence of MK in South India was estimated at 113/100,000/year and in Nepal 799/100,000/year.^7,8^ There is only one older report of the incidence of MK in SSA from Malawi, which suggested a rate of around 180/100,000/year.^9^ Rates in high-income settings are lower.^10^

There are many potential risk factors that may predispose a person to developing MK with some risk factors being more specific to settings (region, income status and organism) and some being ubiquitous. Risk factors such as trauma especially with vegetative matter have been associated with fungal keratitis compared to a pre-existing ocular disease for bacterial keratitis.^11,12^ Injury with mud is strongly linked to Acanthamoeba keratitis.^12^ In addition, agricultural work and foreign body in the eye have been implicated.^11,13,14^ Risk factors that are more setting specific include the use of contact lenses, which affects more people in high-income countries as opposed to the use of traditional eye medicines (TEM), which is more of a problem in Low and Middle-Income Countries (LMIC).^9,15–18^ Other identified risk factors include age (trauma being common in the lower age groups versus ocular surface diseases in older folk), gender (males engaging more in outdoor activities than females) and poverty (MK mostly is more prevalent in among the poor).^11–13^ Diabetes Mellitus (DM) has been the most commonly reported systemic risk factor, especially following keratoplasty or corneal trauma.^19–21^

In the few studies from SSA on the risk factors for MK, suggested factors include trauma and use of TEM.^22–24^ The other risk factors reported in the literature are steroid use, severe staphylococcal eyelid infections and the HIV positive cases.^17,22–25^ However, all previous studies from Africa, had a limited extrapolation of outcome due to the lack of controls.

The aim of this study, therefore, was to investigate the role of multiple risk factors (HIV infection, DM, farming) which are preventable or modifiable by comparing MK cases to disease free community controls, matched for age, sex and village in Uganda.

## Methods

### Ethical statement

This study followed the tenets of the Declaration of Helsinki. It was approved by the London School of Hygiene & Tropical Medicine Ethics Committee (Ref 10647), Mbarara University Research Ethics Committee (Ref 10/04-16) and Uganda National Council for Science and Technology (Ref HS-2303). Written informed consent in Runyankore, the local language, was obtained before enrolment. If the patient was unable to read, the information was read to them, and they were asked to consent by application of the thumbprint which was independently witnessed.

### Study design and setting

A pair-matched case control study was used with a 1:1 case-to-control ratio. The cases were recruited during the main cohort study that prospectively enrolled patients with MK that presented to Ruharo Eye Centre (REC) and Mbarara University and Referral Hospital Eye Centre (MURHEC) from December 2016 to March 2018. MURHEC is a government owned tertiary eye unit established in 2013. It provides mostly free services and attends to about 6,000–10,000 patients/year. REC is a church-based fee-paying tertiary eye hospital founded in the 1960s. Attendance is about 20,000–25,000 patients/year. Both hospitals are in Mbarara Municipality, South-Western Region, Uganda, approximately four hours’ drive far from Kampala. The two units are about 5km apart and work closely. Controls were enrolled in communities where the cases came from.

### Study participants

For the purpose of this study microbial keratitis was defined as the loss of corneal epithelium (of at least 1mm diameter) with underlying stromal infiltrate, associated with any or all signs of inflammation (conjunctival injection, anterior chamber inflammatory cells, ± hypopyon).^26^ Controls were healthy individuals (without any current eye complaint) matched for gender and address. For cases and controls, we excluded those not willing to participate, those not willing to return for follow-up, pregnant women, lactating mothers and those aged below 18 years.

### Sample size

The prevalence of HIV in the general population in Uganda is 6%. A sample of 200 case-control pairs would have 80% power and 95% confidence to detect an odds Ratio of three between cases and controls. From Tanzanian data we expected that perhaps more than 10% of MK cases in our cohort would have HIV infection.^17,27^

### Assessment

#### Cases

We documented baseline demographic information and ophthalmic history including how the eye became infected, predisposing factors such as trauma, prior use of Traditional Eye Medicine (TEM), treatment received, and their “health care seeking journey” before reaching to the eye hospital. In summary, cases underwent a detailed anterior and posterior segment examination on a slit lamp. Corneal scrapes were collected for microscopy, culture and sensitivity and molecular diagnosis. HIV counselling and testing were offered, as per the Uganda Ministry of Health HIV testing protocol where three rapid tests (Determine HIV-1/2/O [Abbott Laboratories, Abbott Park, IL], HIV 1/2 Stat-Pak Ultra-Fast [Chembio Diagnostic Systems, Medford, NY] and Uni-Gold Recombinant HIV-1/2 [Trinity Biotech, Bray, Ireland]) were used to screen participants.^28^ For those who were confirmed as HIV positive, a CD4 test was performed for level of immune suppression. They were referred to the HIV care centre, which is on the hospital site. If a patient refused the HIV test, they were still enrolled for the main cohort but were censored for the nested study. A peripheral prick for blood sugar was taken and WHO guidelines were used to make a diagnosis of Diabetes (random glucose >11.1mmol/L or fasting glucose of >7.0mmol/L).^29^ Cases were treated empirically at presentation; the treatment choice was reviewed after microbiology results according to the hospital protocol. The study follow-up assessment was on day 2, day 7, day 21 and at day 90 to determine their outcome. Additional assessments were conducted as clinically indicated.

#### Controls

At 3 months, the cases were followed-up in their homes for a final assessment at which point healthy community controls were enrolled. Enrolment followed a similar method as previously used in Ethiopia.^30^ The research team visited the villages (100–200 households), the local village head was asked to write down all the eligible controls in that village. They were people of the same gender and in a similar age bracket (decade) as the case. One person was randomly selected from this list using a lottery method, explained to the details of the study and invited to participate if eligible. If a selected control refused or was ineligible, another was randomly selected by lottery. Demographic data was collected as well as a detailed history of exposure to trauma, TEM use, DM and HIV status. A random blood sugar and HIV counselling and testing were offered, as per the Uganda Ministry of Health HIV testing protocol. Home testing for HIV is widely practised in Uganda. For those who were confirmed as HIV positive, a CD4 test was performed for the level of immune suppression. They were referred to the nearest HIV centre for appropriate care. Cases and controls were asked to self-report their wealth status compared to their neighbours using a scale of 1 “very poor” 2 “poor” 3 “neither poor nor rich” 4 “rich” 5 “very rich”.^31^

### Analysis

Data were analysed in STATA v14. All cases and controls were individually matched by age, gender and village. However, we noticed in the analysis that all the pairs had not been correctly matched on age because the village heads had subjectively guessed the ages of the controls. We thus adjusted for age throughout the analysis. We compared the proportions of potential risk factor exposures among cases and control and performed a Mcnemar’s chi^2^ test (binary exposures) and a univariable conditional logistic regression (categorical exposures) for significance of the differences. The main exposures of interest were HIV positive patients, DM patients, farmers and participants with a positive history of trauma and or TEM use.

Multivariable conditional logistic regression analysis was used to estimate odds ratios (ORs) and 95% CI’s of risk factors of MK. The likelihood ratio test was used to assess statistical significance of associations. Variables with a *p*-value less than 0.1 were introduced in the multivariable model. For variables with a high collinearity, the variable of most interest was included in the model. A backward stepwise approach was then used until only the variables with a *p*-value of less than 0.05 were retained.

Because it was not possible to enrol controls for all the cases in the cohort, a separate analysis was performed to compare the baseline characteristics of the cases who had controls and those who did not to look for any systematic bias. A Pearson Chi test (categorical variables) or a Wilcoxon rank sum test (continuous variables) was used to test for significance of the differences.

## Results

A total of 215 controls were enrolled out of 260 eligible cases who had 3-months outcome data. It was not possible to enrol controls for 45 cases because of several reasons. These included: not at home at the agreed time of the home visit (11), wrong home address (4), died (1), uncooperative village members (20), case address too far (9). The cases without controls were dropped from the matched risk factor analysis. We compared the baseline characteristics and exposure proportions between the cases for whom we were able to enrol controls versus the patients without controls (Table 1). Overall, these two groups were comparable across most of the characteristics. However, there was a significant difference in the proportion of HIV (22%) among the cases without controls versus the cases with controls (8%), (Chi-square test 10.7, *p* = .001, df 1). The overall prevalence of HIV was 12% and DM was 7%. Out of all the 37 HIV positive cases, 14 (38%) were newly diagnosed after presenting with MK. They were unaware of their current HIV status or had previously tested negative. The median CD4 count was 358 cells/µL (IQR 267–533, total range 154–1,053). Out of the 22 DM patients, 11 (50%) were diagnosed after presenting with MK.

**Table 1.** Comparison of people who were enrolled into the nested case-control and those who were not (n = 313).

	Enrolled into the case-control (n = 215)			Not enrolled (n = 98) ǂ			
Variable	Median	(IQR)	(Total range)	Median	(IQR)	(Total range)	P value
Age	50	(37–60)	(18–96)	42	(33–59)	(18–87)	.040
Distance	78	(53–120)	(1.5–286)	85	(48–183)	(0.2–378)	.171
Household population	7	(5–8)	(1–28)	6	(3–8)	(1–18)	.030
Distance to nearest Health Centre in KM	3	(1–4)	(0–45)	2	(1–4)	(0–35)	.215
Variable	Category	count	(%)	count	(%)		P value
Gender	Female	101	(47)	38	(39)		.176
	Male	114	(53)	60	(61)		
Occupation	Farmer	157	(73)	63	(64)		.117
	Non-farmer	58	(27)	35	(36)		
Marital status	Not married*	61	(28)	34	(35)		.259
	Married	154	(72)	64	(65)		
Education status	None	60	(28)	24	(25)		.896
	Primary	110	(51)	52	(53)		
	Secondary	31	(14)	14	(14)		
	Tertiary	14	(7)	8	(8)		
Being head of household	Yes	146	(68)	66	(67)		.922
	No	69	(32)	32	(33)		
Being HIV positive (overall 12%) Ɨ	Yes	18	(8%)	19	(22%)		.001
	No	197	(92%)	67	(78%)		
Being a Diabetic patient (overall 7%) Ɨ	Yes	14	(7%)	8	(9%)		.385
	No	201	(93%)	77	(91%)		

*Not married refers to single, separated, divorced or widowed. Ɨ missing results for HIV and diabetes, it was not possible to test everyone for HIV and Diabetes. ǂ These 98 include the 53 that were lost to follow up and the 45 cases with follow-up data at 3 months but to whom controls could not be enrolled.

Table 2 shows exposure comparison among the cases and controls matched for age, sex and village. The proportion of HIV positive patients among the cases was 9% versus 1% among the controls (*p* = .0003). DM was 7% among the cases versus 1.4% among the controls (*p* = .012). Sixty-one (29%) of the cases reported eye trauma before onset of symptoms, none of the controls reported any trauma in the previous 3 months. One hundred and twenty-eight (61%) of the cases reported having used TEM versus only one control who had recently used TEM. Cases more than controls had more people in the poor social economic bracket (*p* = .0001) and lived further from the nearest village health centre, median distance 3km (IQR 1–4, total range 0–45) versus 2km (IQR 1–3, total range 1–15) among the controls (*p* < .0001).

**Table 2.** A matched comparison of exposures among 215 case-control pairs. (gender and village and adjusted for age).

	Cases (215)		Controls (215)		P-value
Exposure	n	(%)	n	(%)	
Married	154	(72)	143	(67)	.215
Head of household	146	(68)	140	(65)	.441
Education status					
None	60	(28)	48	(22)	.148
Primary	110	(51)	114	(53)	
Secondary	31	(14)	32	(15)	
Tertiary	14	(7)	21	(10)	
Farming occupation (if yes)	157	(73)	168	(78)	.144
Trauma (if yes, n = 214)	63	(29)	0	(0)	<.0001
Traditional Eye Medicine (if yes)	133	(62)	1	(0.5)	<.0001
HIV (being positive) *	18	(9)	2	(1)	.0001
Diabetes Mellitus (being positive) Ɨ	14	(7)	3	(1.4)	.012
Size of the household					
Small (1–4 people)	50	(23)	109	(51)	
Medium (5–10 people)	115	(54)	94	(44)	
Large (>11 people)	50	(23)	12	(5)	
Self-reported wealth status ǂ					
Poor	36	(18)	20	(9)	.003
Middle	158	(74)	188	(89)	
Upper	21	(8)	6	(2)	
Type of water source					
Well	103	(50)	107	(52)	
Tap	85	(41)	74	(36)	
Other	17	(9)	25	(12)	
	median	(IQR)	median	(IQR)	
Distance to nearest Health centre	3	(1–4)	2	(1–3)	<.0001

*Twelve cases had missing HIV results, however, all the controls had HIV results reported. Ɨ Nineteen Cases had missing Diabetes test results. self-reported wealth status was classified as poor (1” very poor” 2” poor”), middle (3 “neither poor nor rich”) upper (4 “rich” 5 “very rich”)

Table 3 shows the univariable and multivariable analysis for risk factors of MK adjusted for age, sex and village. These were all adjusted for age, sex and village. In the final model, important risk factors were HIV (OR 83.5, 95%CI 2.01–3456, *p* = .020), DM (OR 9.38, 95% CI 1.48–59.3, *p* = .017), a farming occupation (OR 2.60, 95%CI 1.21–5.57, *p* = .014) and living far from a health facility (OR 1.39, 95%CI 1.14–1.67, *p* = .001) were strongly associated with MK. On the other hand, a middle compared to a low social economic status was less associated with MK (OR 0.29, 95%CI 0.09–0.89), *p* < .0001).

**Table 3.** A matched univariable and multivariable analysis of risk factors of Microbial Keratitis among 215 case-control pairs (matched for sex, village and adjusted for age).

	Univariate Analysis			Multivariable Analysis		
Variable	Crude OR	(95% CI)	p-value	Adjusted OR	(95% CI)	p-value
**Farming occupation** (if yes)	2.10	(1.12–3.92)	.021	2.60	(1.21–5.57)	.014
**HIV** (being positive)	18.3	(2.41–139)	.005	83.5	(2.01–3456)	.020
**Diabetes Mellitus** (being positive)	4.75	(1.29–17.6)	.019	9.38	(1.48–59.3)	.017
Size of the household*						
Small (1–4 people)	1	(reference)	<.0001			
Medium (5–10 people)	5.09	(2.89–8.94)				
Large (>11 people)	1.88	(0.69–5.12)				
**Social economic status**						
Poor	1	(reference)	<.0001	1	(reference)	<.0001
Middle or upper Ɨ	0.21	(0.08–0.56)		0.29	(0.09–0.89)	
Upper	1.14	(0.23–5.58)		1.96	(0.34–10.9)	
**Distance to the nearest Health Centre (increase/km)**	1.32	(1.14–1.53)	<.0001	1.39	(1.14–1.67)	.001

*Family size was highly correlated with wealth status (*p* = 0.02) and was not included in the model. All analysis was adjusted for age

## Discussion

This was the first case control study in SSA to investigate the risk factors of MK. We found that the significant risk factors were trauma, HIV, DM, farming, living far from a health facility and poverty.

The odds of being HIV-positive was higher among MK cases than in the controls, suggesting HIV is a risk factor for MK. HIV affects the immune system making its host susceptible to a range of opportunistic infections. Two previous studies, both from Tanzania, suggested a possible relationship between HIV and Keratitis.^17,25^ In the first study in 1999, the proportion of HIV among MK cases was 40% with a statistically significant trend towards fungal Keratitis.^25^ In the second study in 2003, the proportion of HIV among MK cases was 16%.^17^ Even though anti-retroviral therapy (ART) is now widely available in most parts of SSA, the findings of our study confirmed that HIV is still an independent risk factor for MK. The proportion of HIV positive cases in our cohort was 12%, which was about double the national average of 6.3%.^27^ In the group that was considered for the case control analysis, the proportion of HIV was much lower among the cases (9% as opposed to 12%) and controls (1% as opposed to 6% national average). We speculate that this might have been due to “healthy user bias” where people who thought they were HIV negative were more likely to consent as controls. Although HIV counselling and testing is widely practised in Uganda, almost 40% of the HIV positive patients in this study were identified after presenting with MK. They were unaware their HIV status or previously thought that they were negative.

We found that the possibility of MK is higher in DM. Although this had been suggested from other regions, there have not been any previous studies in SSA that described this association.^32,33^ The proportion of DM among the MK cases was 7%, about thrice the national urban average and seven times the rural average.^34^ DM also affects the immune system making the host susceptible to infection. Additionally, hyperglycaemia provides essential nutrients for the pathogens to thrive. This makes treatment more challenging as these patients tend to respond slowly. The prevalence of DM is on the rise globally due to lifestyle changes, In Uganda, there has been a three fold rise over the last decade.^34^ We have since started offering routine HIV and Diabetes screening for all patients presenting with MK

A farming occupation was another identified risk factor; our hypothesis is that this was linked to trauma. However, trauma could not be tested in the model because of none of the controls reported trauma in the last 3 months. We found that even among the MK cases, trauma rates were lower than anticipated (29%). This is consistent with other studies from sub-Saharan Africa (SSA). In an older study from Ghana, 39% of MK cases reported some form of eye injury prior to onset.^22^ In two separate studies from Tanzania 24% and 39% of cases were associated with trauma.^17,35^ These levels appear to be lower than those reported from South Asia, where the proportion of MK cases associated with an injury is typically around 75%.^11,36,37^ The reason for this difference is not immediately apparent. Perhaps eye trauma was either not as common in SSA as South Asia or it was too subtle to be recalled. This might explain why there were even much less recall among the controls. As one intervention, farmers could be sensitized and encouraged to use eye protection while working.

Poverty and health are intricately related. It is linked to decisions and practises which predisposes individuals to disease, limit access to care and determine choices of treatment options (such as use of TEM). In our study, the odds of MK among individuals of a “low” economic status were about four times more than individuals over a “middle” economic status. According to the latest Uganda household survey, about 30% of the population is poor.^38^ This translates into 12 million who are at an increased risk of MK. In this study, we noticed that cases were more likely to be poorer and live further from the nearest health centre.

### Strengths and limitations

We were not able to enrol controls for all the MK cases in the cohort. However, the sample size was enough to detect important risk factors and enrolling all the controls may have provided minimal addition. Trauma could not be tested as risk factors because there was no reported episode among the controls. Although we were interested in TEM as a risk factor, it was not feasible to test this: we could not ascertain whether it had been applied before or after onset of MK. Before this study, HIV had been suggested as a potential risk factor. This study provided strong evidence of HIV as an independent risk factor for MK, and although the confidence interval is wide, the estimated effect is large. This was the first case control design to investigate risk factors of MK in SSA.

## Conclusion

HIV, DM, a farming occupation and poverty were important risk factors for MK in Uganda. There is need for more work to be done to explore mechanisms of interaction and how these can inform prevention strategies against MK. Patients with MK should be offered HIV and diabetic screening.

## Submission statement

This submission has not been published anywhere previously and that it is not simultaneously being considered for any other publication.

## References

[1] Bennett JE, Dolin R, Blaser MJ. *Mandell, Douglas, and Bennett’s Principles and Practice of Infectious Diseases E-Book*. Amsterdam: Elsevier Health Sciences; 2014:114–115.

[2] Whitcher JP, Srinivasan M. Corneal ulceration in the developing world–a silent epidemic. *Br J Ophthalmol*. 1997;81(8):622–623. doi:10.1136/bjo.81.8.622. 9349145 PMC1722289

[3] Whitcher JP, Srinivasan M, Upadhyay MP. Corneal blindness: a global perspective. *Bull World Health Organ*. 2001;79:214–221. 11285665 PMC2566379

[4] Flaxman SR, Bourne RR, Resnikoff S, et al. Global causes of blindness and distance vision impairment 1990–2020: a systematic review and meta-analysis. *Lancet Global Health*. 2017;5(12):e1221–e1234. doi:10.1016/S2214-109X(17)30393-5. 29032195

[5] Resnikoff S, Pascolini D, Etya’ale D, et al. Global data on visual impairment in the year 2002. *Bull World Health Organ*. 2004;82(11):844–851. doi:S0042-96862004001100009. 15640920 PMC2623053

[6] Marmamula S, Khanna RC, Rao GN. Unilateral visual impairment in rural south India-Andhra Pradesh Eye Disease Study (APEDS). *Int J Ophthalmol*. 2016;9(5):763–767. doi:10.18240/ijo.2016.05.23. 27275437 PMC4886893

[7] Gonzales CA, Srinivasan M, Whitcher JP, Smolin G. Incidence of corneal ulceration in Madurai district, South India. *Ophthalmic Epidemiol*. 1996;3(3):159–166. doi:10.3109/09286589609080122. 8956320

[8] Upadhyay MP, Karmacharya PC, Koirala S, et al. The Bhaktapur eye study: ocular trauma and antibiotic prophylaxis for the prevention of corneal ulceration in Nepal. *Br J Ophthalmol*. 2001;85(4):388–392. doi:10.1136/bjo.85.4.388. 11264124 PMC1723912

[9] Courtright P, Lewallen S, Kanjaloti S, Divala DJ. Traditional eye medicine use among patients with corneal disease in rural Malawi. *Br J Ophthalmol*. 1994;78(11):810–812. doi:10.1136/bjo.78.11.810. 7848973 PMC504961

[10] Erie JC, Nevitt MP, Hodge DO, Ballard DJ. Incidence of ulcerative keratitis in a defined population from 1950 through 1988. *Arch Ophthalmol*. 1993;111(12):1665–1671. doi:10.1001/archopht.1993.01090120087027. 8155038

[11] Bharathi MJ, Ramakrishnan R, Meenakshi R, Padmavathy S, Shivakumar C, Srinivasan M. Microbial keratitis in South India: influence of risk factors, climate, and geographical variation. *Ophthalmic Epidemiol*. 2007;14(2):61–69. doi:10.1080/09286580601001347. 17464852

[12] Bharathi MJ, Ramakrishnan R, Meenakshi R, Shivakumar C, Raj DL. Analysis of the risk factors predisposing to fungal, bacterial & Acanthamoeba keratitis in south India. *Indian J Med Res*. 2009;130:749–757. 20090138

[13] Gandhi S, Shakya D, Ranjan K, Bansal S. Corneal ulcer: a prospective clinical and microbiological study. *Int J Med Sci Public Health*. 2014;3(11):1334–1337. doi:10.5455/ijmsph.

[14] Kursiah MR, Sharif FM, Balaravi P. Retrospective review of corneal ulcers in Ipoh Hospital. *Med J Malaysia*. 2008;63:391–394. 19803298

[15] Houang E, Lam D, Fan D, Seal D. Microbial keratitis in Hong Kong: relationship to climate, environment and contact-lens disinfection. *Trans R Soc Trop Med Hyg*. 2001;95(4):361–367. doi:10.1016/S0035-9203(01)90180-4. 11579873

[16] Bourcier T, Thomas F, Borderie V, Chaumeil C, Laroche L. Bacterial keratitis: predisposing factors, clinical and microbiological review of 300 cases. *Br J Ophthalmol*. 2003;87(7):834–838. doi:10.1136/bjo.87.7.834. 12812878 PMC1771775

[17] Burton MJ, Pithuwa J, Okello E, et al. Microbial keratitis in East Africa: why are the outcomes so poor? *Ophthalmic Epidemiol*. 2011;18(4):158–163. doi:10.3109/09286586.2011.595041. 21780874 PMC3670402

[18] Ong HS, Fung SS, Macleod D, Dart JK, Tuft SJ, Burton MJ. Altered patterns of fungal keratitis at a London ophthalmic referral hospital: an eight-year retrospective observational study. *Am J Ophthalmol*. 2016;168:227–236. doi:10.1016/j.ajo.2016.05.021. 27287820 PMC4977014

[19] Nath R, Baruah S, Saikia L, Devi B, Borthakur AK, Mahanta J. Mycotic corneal ulcers in upper Assam. *Indian J Ophthalmol*. 2011;59(5):367–371. doi:10.4103/0301-4738.83613. 21836342 PMC3159318

[20] Sengupta J, Khetan A, Saha S, Banerjee D, Gangopadhyay N, Pal D. Candida keratitis: emerging problem in India. *Cornea*. 2012;31(4):371–375. doi:10.1097/ICO.0b013e31823f8a71. 22262225

[21] Bharathi MJ, Ramakrishnan R, Vasu S, Meenakshi R, Palaniappan R. Epidemiological characteristics and laboratory diagnosis of fungal keratitis. A three-year study. *Indian J Ophthalmol*. 2003;51:315–321. 14750619

[22] Hagan M, Wright E, Newman M, Dolin P, Johnson G. Causes of suppurative keratitis in Ghana. *Br J Ophthalmol*. 1995;79(11):1024–1028. doi:10.1136/bjo.79.11.1024. 8534648 PMC505322

[23] Ezegwui IR. Corneal ulcers in a tertiary hospital in Africa. *J Natl Med Assoc*. 2010;102(7):644–646. doi:10.1016/S0027-9684(15)30642-8. 20690328

[24] Oladigbolu K, Rafindadi A, Abah E, Samaila E. Corneal ulcers in a tertiary hospital in Northern Nigeria. *Ann Afr Med*. 2013;12(3):165–170. doi:10.4103/1596-3519.117626. 24005589

[25] Mselle J. Fungal keratitis as an indicator of HIV infection in Africa. *Trop Doct*. 1999;29(3):133–135. doi:10.1177/004947559902900303. 10448232

[26] Prajna NV, Jeena M, Tiruvengada K, et al. Comparison of natamycin and voriconazole for the treatment of fungal keratitis. *Arch Ophthalmol*. 2010;128(6):672–678. doi:10.1001/archophthalmol.2010.102. 20547942 PMC3774126

[27] International. UMoHaI. *2011 Uganda AIDS Indicator Survey: Key Findings*. MOH, ed. Calverton, Maryland, USA: MOH and ICF International; 2012.

[28] Gray RH, Makumbi F, Serwadda D, et al. Limitations of rapid HIV-1 tests during screening for trials in Uganda: diagnostic test accuracy study. *Bmj*. 2007;335(7612):188. 17545184 10.1136/bmj.39210.582801.BE PMC1934458

[29] Organization WH. Definition and diagnosis of diabetes mellitus and intermediate hyperglycaemia: report of a WHO/IDF consultation. 2006.

[30] Habtamu E, Wondie T, Aweke S, et al. The impact of trachomatous trichiasis on quality of life: a case control study. *PLoS Negl Trop Dis*. 2015;9(11):e0004254. doi:10.1371/journal.pntd.0004254. 26598937 PMC4657886

[31] Habtamu E, Wondie T, Aweke S, et al. Trachoma and relative poverty: a case-control study. *PLoS Negl Trop Dis*. 2015;9(11):e0004228. doi:10.1371/journal.pntd.0004228. 26600211 PMC4657919

[32] Rosa RH, Miller D, Alfonso EC. The changing spectrum of fungal keratitis in south Florida. *Ophthalmology*. 1994;101(6):1005–1013. doi:10.1016/S0161-6420(94)31225-5. 8008340

[33] Weissman BA, Mondino BJ. Risk factors for contact lens associated microbial keratitis. *Cont Lens Anterior Eye*. 2002;25(1):3–9. doi:10.1016/S1367-0484(01)00002-9. 16303475

[34] Bahendeka S, Wesonga R, Mutungi G, Muwonge J, Neema S, Guwatudde D. Prevalence and correlates of diabetes mellitus in Uganda: a population‐based national survey. *Trop Med Int Health*. 2016;21(3):405–416. doi:10.1111/tmi.2016.21.issue-3. 26729021

[35] Poole TR, Hunter DL, Maliwa EM, Ramsay AR. Aetiology of microbial keratitis in northern Tanzania. *Br J Ophthalmol*. 2002;86(8):941–942. doi:10.1136/bjo.86.8.941. 12140229 PMC1771226

[36] Srinivasan M, Gonzales CA, George C, et al. Epidemiology and aetiological diagnosis of corneal ulceration in Madurai, south India. *Br J Ophthalmol*. 1997;81(11):965–971. doi:10.1136/bjo.81.11.965. 9505820 PMC1722056

[37] Chidambaram JD, Venkatesh Prajna N, Srikanthi P, et al. Epidemiology, risk factors, and clinical outcomes in severe microbial keratitis in South India. *Ophthalmic Epidemiol*. 2018;25(4):297–305. doi:10.1080/09286586.2018.1454964. 29580152 PMC5985925

[38.] Statistics UBo. *Uganda National Household Survey, 2016/2017: socio-economic Module*. Vol. 6. Kampala: Uganda Bureau of Statistics; 2018.

